# Development, in vitro validation and human application of a novel method to identify arrhythmia mechanisms: The stochastic trajectory analysis of ranked signals mapping method

**DOI:** 10.1111/jce.13882

**Published:** 2019-03-05

**Authors:** Shohreh Honarbakhsh, Ross J. Hunter, Malcolm Finlay, Waqas Ullah, Emily Keating, Andrew Tinker, Richard J. Schilling

**Affiliations:** ^1^ Electrophysiology Department The Barts Heart Centre, Barts Health NHS trust London United Kingdom; ^2^ William Harvey Research Institute, QMUL

**Keywords:** atrial fibrillation, atrial tachycardia, catheter ablation, mapping method, optical mapping

## Abstract

**Introduction:**

Stochastic trajectory analysis of ranked signals (STAR) is a novel method for mapping arrhythmia. The aim was to describe its development and validation as a mapping tool.

**Methods and Results:**

The method ranks electrodes in terms of the proportion of the time they lead relative to neighboring electrodes and ascribes a predominant direction of activation between electrodes. This was conceived with the aim of mapping atrial fibrillation (AF) drivers. Validation of this approach was performed in stages. First, in vitro simultaneous multi‐electrode array and optical mapping were performed on spontaneously fibrillating HL1 cell cultures, to determine if such a method would be able to determine early sites of activation (ESA). A clinical study acquiring unipolar electrograms using a 64‐pole basket for the purposes of STAR mapping in patients undergoing atrial tachycardia (AT) ablation. STAR maps were analyzed by physicians to see if arrhythmia mechanisms could be correctly determined. Mapping was then repeated during atrial pacing. STAR mapping of in vitro activation sequences accurately correlated to the optical maps of planar and rotational activation. Thirty‐two ATs were mapped in 25 patients. The ESA accurately identified focal/micro‐reentrant ATs and the mechanism of macro‐reentrant ATs was effectively demonstrated. STAR method accurately identified four pacing sites in all patients.

**Conclusions:**

This novel STAR method correlated well with the gold standard of optical mapping in vitro and was able to accurately identify AT mechanisms. Further analysis is needed to determine whether the method might be of use mapping AF.

## 1 INTRODUCTION

The Stochastic Trajectory Analysis of Ranked signals (STAR) is a novel‐mapping method. The method was developed with the specific aim of identifying organized activity in AF, but may also be useful as an adjunct to the conventional mapping of atrial tachycardias (ATs). It was conceived for use in conjunction with whole‐chamber basket catheters to allow for simultaneous global atrial mapping. Due to the variability of AF cycle length (CL) it is not feasible to determine early sites of activation (ESA) in relation to a fixed reference point. For this reason, the STAR mapping method compares activation times of unipolar electrograms in a dynamic fashion across all basket catheter electrode poles. There are essentially two components incorporated in the method: (1) stochastic trajectory analysis, whereby the predominant direction of wavefront activation was determined; and (2) ranking of signals, to determine how often the electrograms at each electrode led relative to its neighbors.

The STAR mapping method was validated in vitro using multi‐electrode arrays (MEAs) and comparing this to the simultaneous optical mapping of calcium transit in HL1 cells. It was then validated in vivo in patients with (i) atrial paced beats in sinus rhythm and (ii) AT where the mechanism was confirmed with conventional mapping, entrainment, and ablation response.

## 2 METHODS

The methods are divided into a description of the STAR mapping method, followed by the (A) in vitro study and (B) clinical study.

### 2.1 STAR mapping method principals

**Figure 1:**
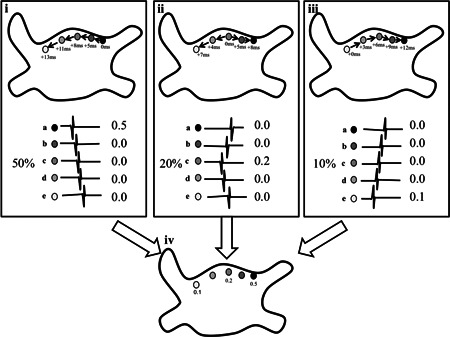
i‐iv, Shows a simplified method of calculating a STAR map. Five electrogram pairs are shown, (a‐e), and the electrograms derived from each electrode are shown below. Three representative patterns which together comprise 80% of the total recording time are shown (i‐iii): (i) accounting for 50% of the time electrode a is leading all other electrodes, (ii) a different activation sequence is illustrated, with electrode (c) leading, accounting for 20% of the time, (iii) is another activation sequence where electrode (e) leads, 10% of the time. Each electrode has a value associated with it based on the proportion of time that electrode is seen as "leading" its closest associated electrode as shown in (iv). A final process combines these data and superimposes these on a combined map, highlighting the leading electrodes. STAR, stochastic trajectory analysis of ranked signals

The principle of STAR mapping is to use data from multiple individual wavefront trajectories to identify regions of the atrium that most often precede activation of neighboring areas. By gathering data from many hundreds of activations, a statistical model can be formed. This permits regions of the atrium to be ranked according to the amount of time that activations precede those of adjacent regions (Figure 1i‐iv).

#### 2.1.1 Electrode pairing and geodesic distance

In addition to the statistical analysis of multiple activations, the geodesic distance between compared regions was limited. The intention was to minimize the chance of ascribing a relationship between two regions activated by unrelated wavefronts, and instead assign relationships only to true sequential activations.

**Figure 2:**
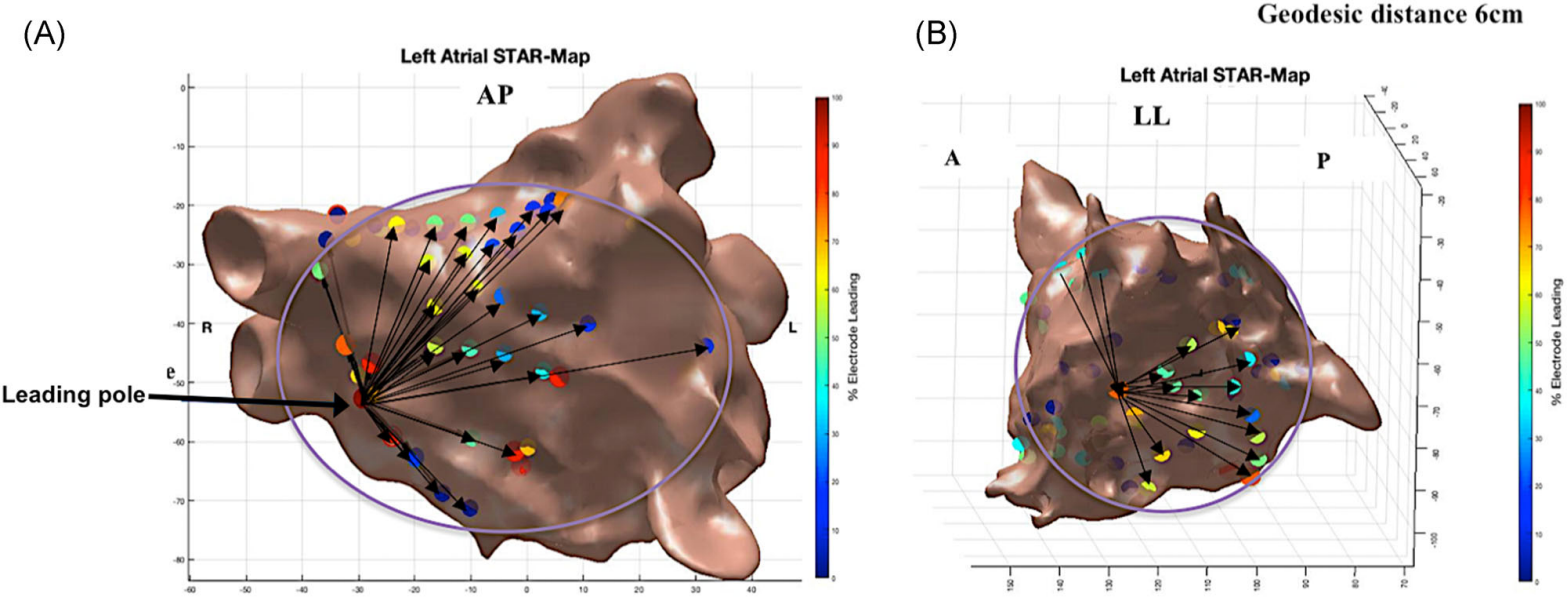
A, A STAR LA map in an anterior‐posterior view (GEPD = 6 cm) demonstrating a focal AT mapped to the septum. B, STAR LA map in a left lateral view (GEPD = 6 cm) demonstrating a mitral isthmus‐dependent AT whereby electrodes anteriorly are leading electrodes posteriorly, as a result, no electrode is leading 100% of the time as seen with a focal AT. AT, atrial tachycardia; GEPD, geodesic electrode pairing distance; STAR, stochastic trajectory analysis of ranked signals

AF drivers have been shown to occupy small discreet locations,^1^, ^2^ distributed throughout the left atrium (LA)^3^, ^4^ with patients having potentially more than one driver.^1^, ^3^ Limiting the maximal electrode separation to be analyzed to a relatively short geodesic distance may be appropriate for the analysis of AF as it allows the LA to be subdivided into sections whereby all electrodes are mapping the same surface that is activated by a driver. In contrast, when mapping an AT with a focal/micro‐reentrant mechanism, only one driver site would be expected, with a longer CL and a 1:1 relationship with the rest of the atria, so electrode pairing could be performed over a larger distance. This ensures that the leading electrode pairs sequentially with all electrodes mapping the widest area possible. It would be expected that a focal/micro‐reentrant AT would appear on a STAR map as the only site whose activation precedes all other electrodes (Figure 2A).

Macro‐reentrant circuits, in contrast, will not display single areas of early activation. On the STAR map produced in such a case, no electrode will lead all others, all the time. If during macro‐reentrant ATs, STAR maps are created using longer geodesic distances each electrode will be paired with an electrode either ahead or behind the wavefront (Figure 2B). The percentage of cycles that an electrode is leading relative to its peers will be dependent on the number of electrodes it is paired with ahead and behind of the wavefront, which in turn is influenced by catheter position, orientation and the limitations placed on geodesic electrode pairing distance (GEPD). No sites will be seen to lead 100% of the time. Adjusting the GEPD should thereby allow STAR maps to aid in defining fibrillatory, focal/micro‐reentrant and macro‐reentrant tachycardias. The effect of different GEPD on the STAR maps produced by different tachycardia mechanisms was compared.

#### 2.1.2 Activation times and pole leaders

Unipolar activation timing was taken as the maximum negative deflection (peak negative dv/dt). Comparing the activation times obtained, the electrodes in the pair can either be labeled as a follower or a leader depending on activation time differences. For every electrode, its overall propensity to be a leading electrode is derived based on the proportion of times it was a leading electrode in every pair. Thus, an electrode can only be a 100% leader if it is activated ahead of all electrodes it is paired with. Conversely, an electrode will be leading 0% of the time if it is consistently following the other electrodes it is paired with.

Filtering using refractory periods was performed with conservative values for a minimum atrial refractory period used (70 ms).^5^, ^6^ If an activation fell within this nominated refractory period it was not considered representative of a separate wavefront this was particularly to avoid fractionated electrograms being labeled as separate activations. Electrode timing relationships that were implausible due to conduction velocity (CV) restraints were discarded. It has been established that CV varies in areas of non‐low voltage zones (LVZs) compared to LVZs. It has also been shown that CV has a negative correlation between bipolar voltage and proportion of non‐LVZs.^7^, ^8^ CVs of 1.59 and 0.98 m/s^7^, ^8^ were used depending on the proportion of non‐LVZs and LVZs that was present, respectively.

#### 2.1.3 Creating and interpreting a STAR map

Color‐coded electrode positions were projected on to patient's LA geometry representation. Colors represented the proportion of time spent leading in relation to the other paired electrodes. Further visual cues highlighting the importance of sites with high‐leading proportions included size scaling of electrode sites and arrowhead lines highlighting wavefront direction.

##### In vitro study

HL1 cells which are immortal murine cardiac atrial cell lines (Sigma‐Aldrich Inc, NM) were used for the in vitro study. HL1 cells were used as they proliferate in culture while maintaining a cardiac myocyte phenotype and contractile activity.^9^ Using these cells it allowed the mapping of spontaneous atrial activation and allowed the comparison of optical maps generated to those created with the STAR mapping method. This was to ensure that the STAR maps could effectively identify ESA and elicit rotational mechanisms as shown on the optical maps. The preparations of the HL1 cells and MEAs are detailed in Supplemental Method.

The MEA was placed under the microscope to allow simultaneous optical mapping and electrical activity recording. Atrial signals were recorded by the 60 electrodes on the MEA and processed through the Multi‐channel rack recording system. They were filtered between 50 to 200 Hz with a sampling frequency of 5000 Hz. Each electrode channel on the recording system was numbered in accordance with the predefined number for each electrode from which the electrical signals were recorded. Manufacturers electrode numbering was used and reviewing the position of these electrodes on the MEA locational data (x and y coordinates) were determined for all the electrodes. The electrical signals obtained and the locational data for each electrode was processed through the STAR mapping method to create 2D STAR maps. The activation times for each electrode was determined using the same principles discussed above. The refractory period and CV used by the STAR mapping method was adjusted to those compatible to that what has been reported in HL1 cells.^10^, ^11^

To visualize calcium movement a calcium sensitive dye (Fluro4) was applied to the HL1 cells and with camera imaging and processing through the ImageJ system optical maps imaging calcium transit were created. Using the optical maps the origin sites of cell activity was determined. These findings were compared to the STAR mapping results. These experiments were repeated using 10 different MEAs with three separate 30‐second recordings being performed using each MEA. This was to allow recordings to be obtained for different wavefront activations which can then be used for the STAR mapping method validation.

##### Clinical study

Patients undergoing persistent AF ablation (<24 months; patients that organized into AT during ablation) and AT (de‐novo or post previous AF ablation) were included. Procedures were performed on uninterrupted anticoagulation, heparin bolusing to maintain an ACT of 300 to 350 seconds, and either conscious sedation or general anesthesia according to physician and patient preference. All patients provided informed consent for their study inclusion, and ethical approval was granted by the UK National Research Ethics System (London‐Bloomsbury Research Ethics Committee, 16/LO/1379). The study was prospectively registered on clinicaltrials.gov (NCT02950844).

##### Electrophysiology study

Mapping was performed with the CARTO3 mapping system (Biosense Webster, Inc, CA). An LA geometry and high‐density bipolar voltage map was created in all patients using a 2 to 6‐2 mm spacing PentaRay NAV catheter (Biosense Webster). The color fill threshold was set at 5 mm. A Thermocool© SmartTouch Surround Flow catheter (Biosense Webster) was used for ablation and atrial pacing.

A 60 or 50 mm 64‐pole basket catheter (Constellation, Boston Scientific, Natick, Minnetoka, MA or FIRMap Abbott, CA) was positioned in the LA through an 8.5Fr SL1 sheath (Daig Medical, MN) under fluoroscopic guidance. Sizing was determined by pre‐procedure transthoracic echocardiogram data. The catheter was repositioned to provide optimal and stable chamber coverage, with minimal interspline bunching.

Unipolar signals were recorded through the Bard electrophysiological recording system (Labsystem Pro, Boston Scientific, Ltd, NA) by reference to a decapolar catheter (Biosense Webster) positioned in the IVC and filtering between 0.5 to 500 Hz. If the coronary sinus (CS) activation was suggestive of a right‐sided AT the right atrium (RA) was also mapped with the basket catheter.

Patients who attended in sinus rhythm first underwent fixed CL atrial pacing before AT induction through decremental burst pacing. Patients attending in AT had their arrhythmia mapped, followed by ablation and subsequently fixed‐length pacing in sinus rhythm. It was felt that to effectively validate the STAR mapping method it required the use of an atrial rhythm where the mechanism could be elicited using conventional mapping.

##### Validation with atrial pacing in sinus rhythm

Atrial pacing was performed in sinus rhythm at 600 ms from four pacing sites (endocardial proximal and distal CS, LA roof and LA appendage), with 30‐seconds of recording per site, and separate STAR maps were created for each pacing site. Two operators reviewed all STAR maps during the case. The maps were also reviewed offline independently by two blinded observers to determine if they were able to identify the pacing site from the STAR maps. In addition, the electrode with the earliest activation on the basket catheter was identified from the electrograms obtained from Bard. The STAR maps were reviewed to ensure that the site of the leading electrode correlated to this electrode. Further to this, the CARTO3 maps were reviewed to ensure the electrode with the earliest activation on the STAR map correlated to the electrode closest to the pacing site.

##### Validation in AT

A minimum of two separate maps was created in all patients during AT, with the basket catheter in different positions. These were compared with the confirmed AT mechanisms, as determined by CARTO3 local activation time (LAT) maps and entrainment, with confirmation of mechanism given by the ablation response. If further ATs were induced following ablation, further mapping and ablation were performed as per protocol. The STAR maps were reviewed prospectively by the two operators but also reviewed independently by two blinded observers to ensure they were able to accurately determine the mechanism of the AT from only the STAR maps.

##### Impact of different GEPD

The maximum GEPD was varied between 1 and 9 cm for all STAR maps. For each GEPD the number of paired electrodes was manually determined from all the maps for each anatomical LA surface using a previously described LA model.^12^ In brief, the LA was segmented into five anatomical surfaces (anterior, roof, septum, lateral, and posterior‐inferior). We also confirmed that paired electrodes were located on the same anatomical surface for each GEPD and that increasing the distance did not result in inappropriate electrode pairing. The average surface area enclosed by all the electrode pairs constituting an island was also determined using a Matlab custom written script.

### 2.2 Statistical analysis

Statistical analyses were performed using SPSS (IBM SPSS Statistics, Version 24 IBM Corp, NY). Continuous variables are displayed as mean ± standard deviation (SD) or median (interquartile range). Categorical variables are presented as a number and percentage. Spearman's Rho correlation coefficient was determined to assess the correlation between GEPD and the surface area and a number of segments mapped. A *P* < 0.05 was deemed significant.

## 3 RESULTS

### 3.1 In vitro study

**Figure 3:**
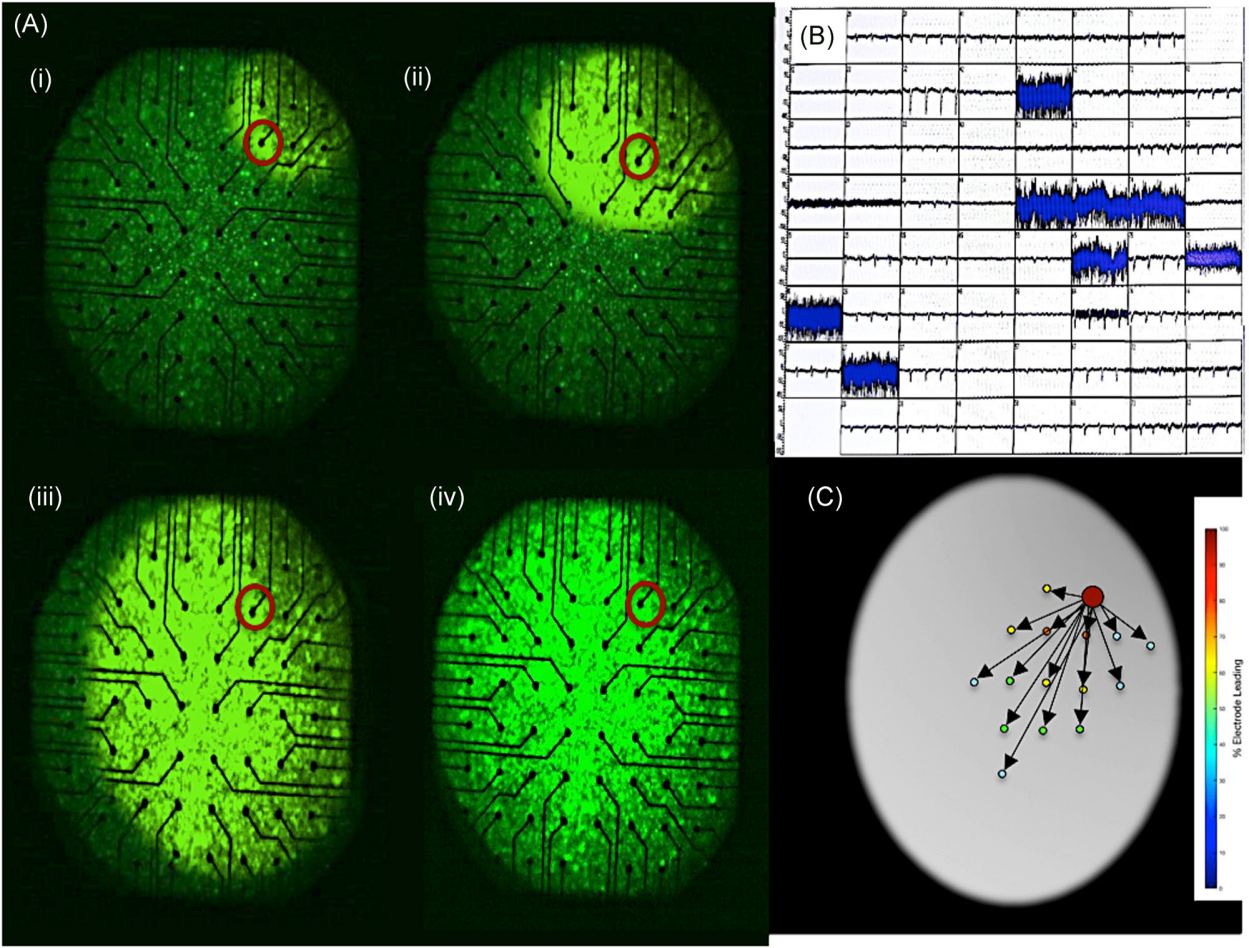
A‐D, (Ai‐iv) 2D optical map demonstrating a planar wavefront moving from the top left corner of the map with the electrode that recorded the earliest activation highlighted with a red circle. B, electrograms recorded by the electrodes on the MEA, of note it highlights the noise experienced on the electrodes that were excluded from the analysis. C, 2D STAR map that shows the earliest activation at the electrode highlighted on the optical map and also shows the wavefront moving across the MEA as seen on the optical map. MEA, multi‐electrode array; STAR, stochastic trajectory analysis of ranked signals

Ten MEAs were used for electrical recordings and optical mapping. Three 30‐second recordings were performed for each of the MEAs leaving a total of 30 electrogram recordings and 30 2D optical maps. Out of these 30 optical maps, 22 demonstrated planar wavefronts and 8 demonstrated rotational wavefronts. Six out of the 60 electrodes had persistent noise on the channel and were therefore excluded leaving 54 electrodes that were used for the analysis. The noise on these electrodes was found to be due to a fault with the recording pins. Processing the electrical signals through STAR mapping allowed the creation of 30 2D STAR maps. All STAR maps indicated the direction of the wavefront and showed the electrode(s) as leading consistently when they were recording the first electrical signals based on the electrical recordings and optical maps (Figure 3A‐C). Further to this, STAR mapping was also able to create maps that allowed the identification of rotational wavefronts as identified on the optical maps.

### 3.2 Clinical study

#### 3.2.1 Patients and procedures

Twenty‐five patients were included (10 persistent AF patients that organized into an AT during ablation and 15 AT patients). Baseline characteristics are demonstrated in Table S1. No complications occurred in any of the patients. During a follow‐up of 13.7 ± 1.3 months, all patients had remained in sinus rhythm with no AT recurrence and off antiarrhythmic drugs.

One patient had limited STAR maps created due to suboptimal LA coverage and contact with the basket catheter in the context of a severely dilated LA (area 39 cm^2^). This patient was thereby excluded leaving 24 patients in the study. A total of 164 STAR maps were created in these patients (6.8 ± 1.4 maps per patient), of which 68 maps were created during AT (2.8 ± 1.4 maps per patient). A majority of these maps were created in the LA (n = 145, 88%).

#### 3.2.2 Atrial pacing validation

**Figure 4:**
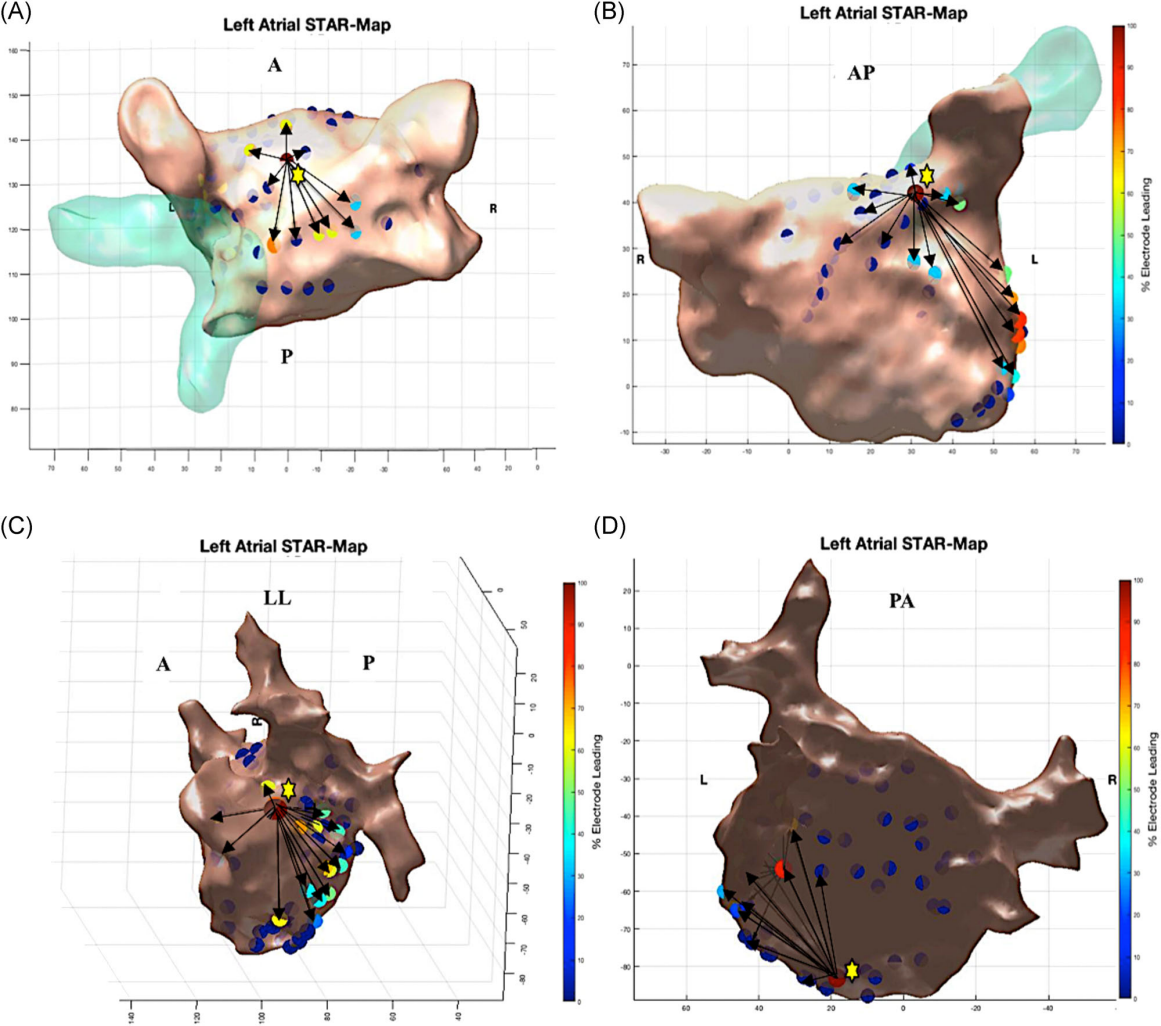
A‐D, Demonstrates STAR maps during atrial pacing at 600ms in sinus rhythm at four different LA sites. A, LA roof. B, LA appendage. C, Endocardial distal CS distal. D, Endocardial proximal CS. The maps show the earliest site of activation near the pacing site as indicated by the yellow star. The STAR mapping method also implements arrows to demonstrate the direction of wavefront propagation. CS, coronary sinus; STAR, stochastic trajectory analysis of ranked signals

Atrial pacing during sinus rhythm at the four sites in the LA (endocardial proximal and distal CS, LA roof and LA appendage) was completed in 22 patients. In two patients consistent capture was not feasible at the LA appendage despite maximum output and consequently pacing was performed at the anteroseptum. Ninety‐six STAR maps were created with atrial pacing (4.0 maps per patient). All STAR maps effectively identified the pacing sites in all patients as confirmed by the two operators and two‐blinded observers (Figure 4A‐D). In all cases, on reviewing the raw unipolar electrograms, the electrode that recorded the earliest manually timed atrial electrogram was confirmed as the electrode(s) that was identified as the leader on the STAR map and was the electrode(s) closest to the pacing site.

#### 3.2.3 Atrial tachycardias

In the 24 patients, 32 ATs were mapped using the STAR mapping method (1.3 ± 0.5 ATs per patient). Of these, 13 ATs resulted from an organization of persistent AF into AT during the catheter ablation procedure. The remaining 19 ATs were mapped in the 14 patients coming into the catheter lab in AT (1.4 ± 0.5 ATs per patient).

**Figure 5:**
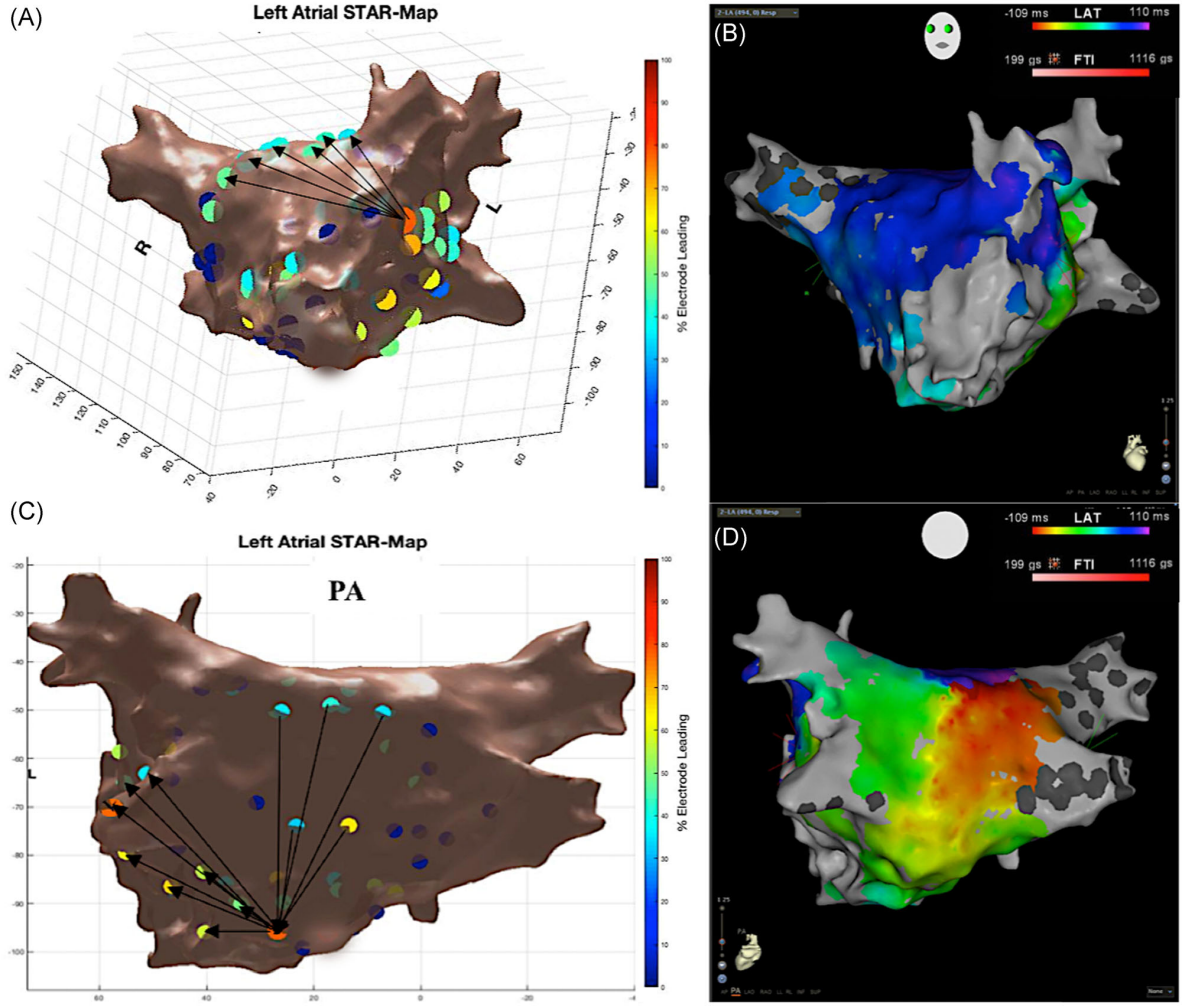
A‐D, Roof‐dependent flutter mapped with STAR and CARTO3 LAT maps. A, STAR map in a tilted LAO view that demonstrates the wavefront moving up the anterior wall, as indicated by the arrows that correlate to B, the LAT map in a tilted LAO view. C, STAR map in posterior‐anterior view that demonstrates the wavefront moving down the posterior wall that is supported by D, the LAT map. LAT, local activation time; STAR, stochastic trajectory analysis of ranked signals

**Figure 6:**
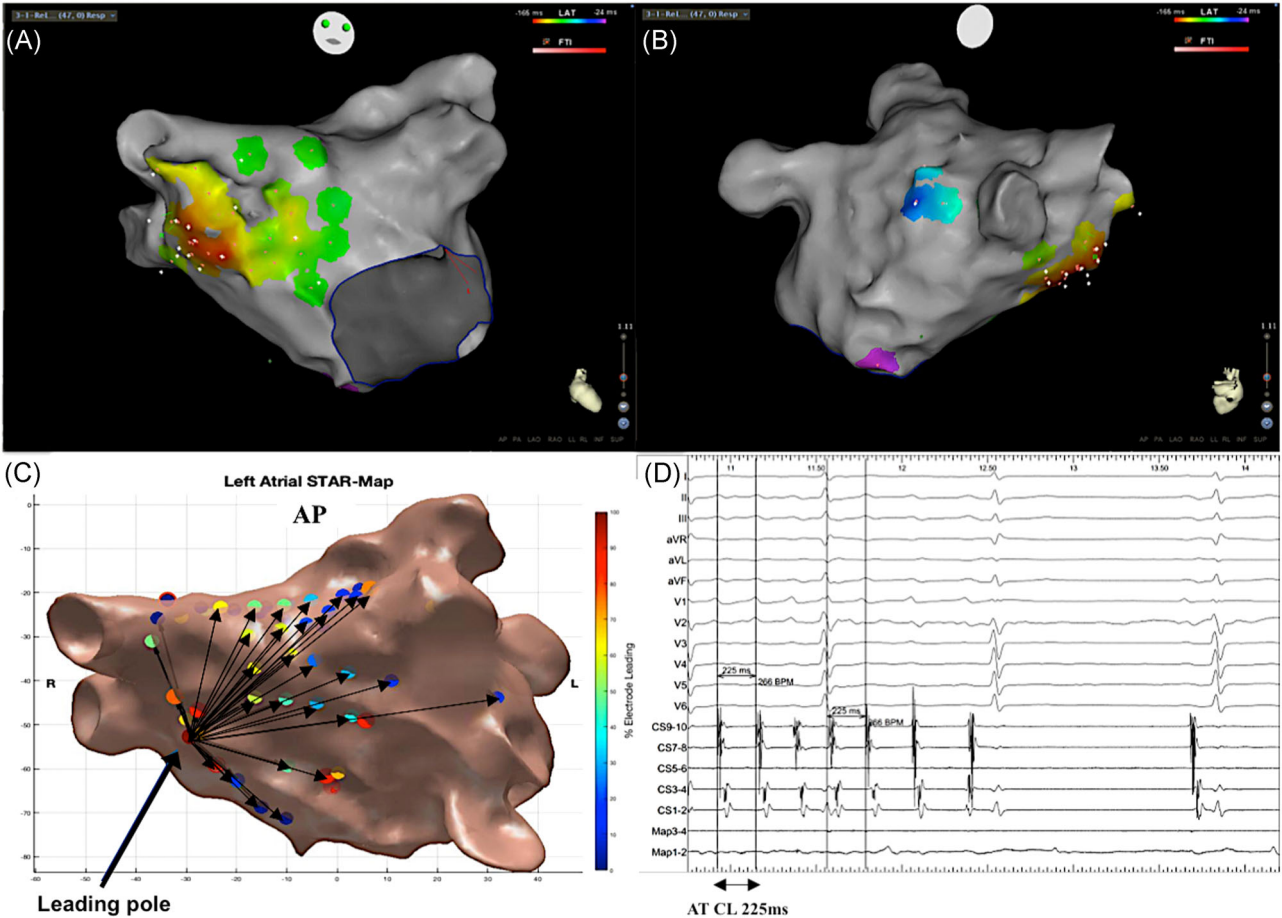
A‐D, Focal/micro‐reentrant AT mapped with STAR and CARTO3 LAT maps. A, LAT map in a titled anterior‐posterior view demonstrating earliest activation at the septum. B, LAT map in a titled right lateral view supporting the spread of the AT from the septum across the LA. C, STAR map that shows the earliest site of activation at the septum that correlates to the focus of the AT. D, BARD electrograms that include the surface ECGs, CS, and map electrograms that shows an AT with a CL of 225 ms terminating to sinus rhythm on ablation at the earliest site of activation. AT, atrial tachycardia; LAT, local activation time; STAR, stochastic trajectory analysis of ranked signals

**Figure 7:**
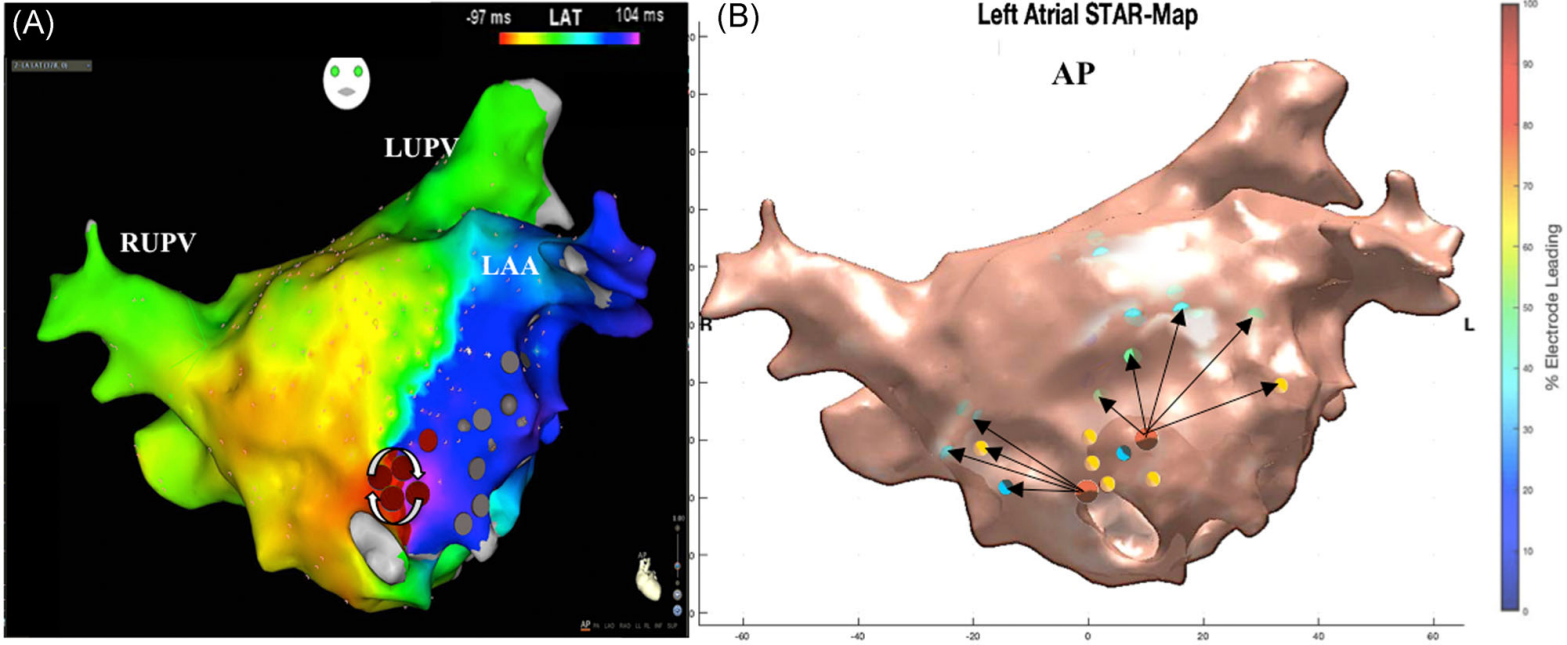
A‐B, Micro‐reentrant AT mapped with STAR and CARTO3 LAT map. A, LAT map in a titled anterior‐posterior view demonstrating a micro‐reentrant AT mapped to the low anterior wall with the arrows further highlighting the mechanism. B, STAR map in an anterior‐posterior view that shows two early sites of activation at the low anterior wall. Neither of these sites is leading 100% of the time as the electrodes closer to the circuit are leading these electrodes. The arrows demonstrate that the wavefront propagates away from these sites across the LA. AT, atrial tachycardia; LAT, local activation time; STAR, stochastic trajectory analysis of ranked signals

**Figure 8:**
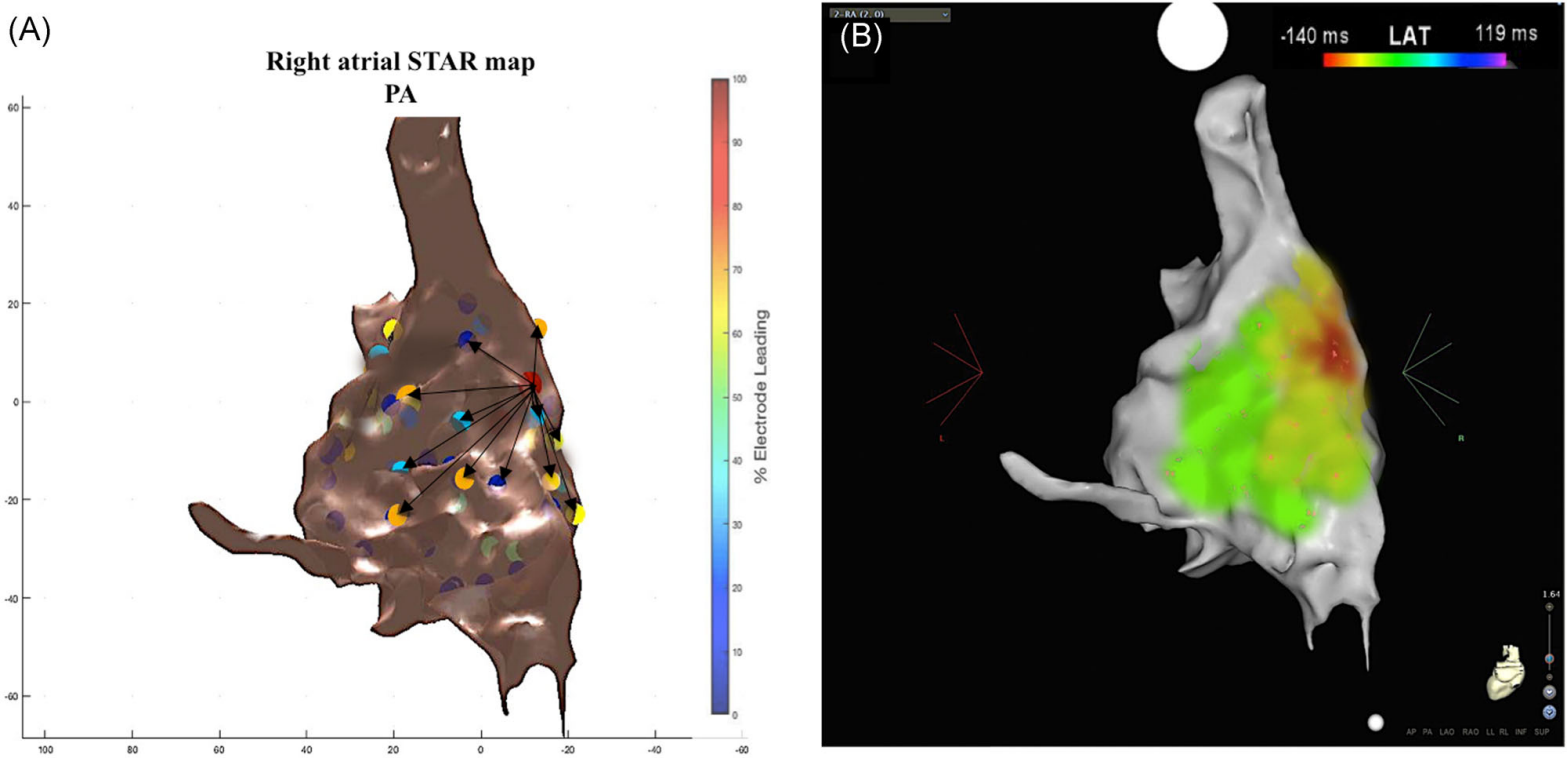
A‐B, STAR and CARTO 3 LAT maps of a focal right‐sided AT. A, A right atrial STAR map in anterior‐posterior view that demonstrates an early site of activation at the right atrial septum with the arrows highlighting the wavefront propagation from this site. B, LAT map of the focal AT. AT, atrial tachycardia; LAT, local activation time; STAR, stochastic trajectory analysis of ranked signals

The mechanism of all 32 ATs were confirmed with conventional LAT maps, entrainment and ablation response. None of the clinical ATs were reinducible during burst atrial pacing post ablation. The ATs mapped included macro‐reentrant (Figure 5A‐D) and micro‐reentrant/focal ATs (Figure 6A‐C, Figure 7A and 7B, and Figure 8A and 8B) that was predominantly left‐sided (n = 25, 78%) (Table 1).

**Table 1:** Mechanism of the ATs mapped

ATs mapped and ablated	
AT, n, %	32
Macroreentrant	21 (65.6)
Mitral isthmus‐dependent flutter	6 (28.6)
Roof‐dependent flutter	9 (42.9)
Cavo‐tricuspid isthmus‐dependent flutter	6 (28.6)
Focal/micro re‐entrant	11 (34.4)
LA low anterior	1 (9.1)
LA mid roof	3 (27.3)
LA low anteroseptal	3 (27.3)
Right focal/microreentrant	1 (9.1)
Ligament of marshall	3 (27.3)

*Note*. AT, atrial tachycardia.

The two operators reviewed all STAR maps created live during the case and agreed in all cases that the STAR maps effectively demonstrated the mechanism of the AT. The two blinded observers independently reviewed the STAR maps after the case whilst blinded to the other procedural data and both were able to determine the AT mechanism in all cases based on the STAR maps alone.

The ablation site in the focal/micro‐reentrant ATs, which resulted in the termination of the tachycardia was within of 0.6 ± 0.2 cm from the leading electrode identified on the STAR map and was within 10 mm in all cases.

#### 3.2.4 Impact of different GEPD

As the GEPD was increased it resulted in a smaller proportion of electrode pairs that only included electrode that mapped the same anatomical surface (0% with GEPD of 9 cm vs 100% with GEPD of less than and equal to 5 cm; Table S2). As one might expect, a larger surface area of the LA was mapped by all electrode pairs when using greater GEPD (8.8 ± 2.4 cm^2^ GEPD = 9 cm vs 0.7 ± 0.5 cm^2^ GEPD = 1 cm). There was a strong positive correlation between the increase in GEPD and surface area (*r*_*s*_ = 1.0; *P* < 0.001). Using very small GEPD resulted in the electrode pairings to include electrodes that only mapped the same anatomical surfaces but it also resulted in each anatomical surface to be segmented into multiple smaller mapped segments or “plaques,” thereby increasing the number of segments over which activation times could be compared (4.6 ± 1.1 anterior mapping segments GEPD = 1 cm vs ≤1.5 anterior mapping segments GEPD ≥ 4 cm). There was a strong negative correlation between the increase in GEPD and the number of mapping segments (*r*_*s*_ = −0.97; *P* < 0.001). However, when segmenting a surface into plaques this gave the artificial appearance of a leading edge when that segment is considered in isolation. GEPD of 3 to 5 cm ensured a good balance of mapping the same anatomical surface without over segmenting it.

## 4 DISCUSSION

This study describes a novel‐mapping method. This was initially validated in vitro using a novel cell preparation and effectively demonstrated both planar and rotational activation. The method was then used in vivo to map atrial paced beats and ATs. The methodology used in this method differs to that used in commercially available mapping systems.^4^, ^13^ The STAR mapping method does not require a window of interest and does not focus on identifying any one particular arrhythmia mechanism, but instead aims to identify predominant wavefront direction and sites that lead relative to surrounding areas. This alternative mapping method was able to demonstrate both macro‐reentry and focal/micro‐reentry ATs.

### 4.1 In vitro validation

Optical mapping provides high‐resolution imaging, is considered a gold standard for the mapping arrhythmias and has demonstrated the presence of rotors during AF in isolated sheep hearts.^14^ In our study, optical maps imaging calcium transit in HL1 cells demonstrated both planar and rotational wavefronts. Utilizing the unipolar electrograms recorded simultaneously with these optical maps, STAR maps were able to show identical arrhythmia mechanisms as on the optical maps.

### 4.2 Pacing validation

STAR mapping was validated using paced atrial beats from multiple sites in the LA. Operators and blinded observers were able to determine the pacing site from these maps, and the electrodes having the highest propensity for leading (as per the STAR mapping method) correlated with the electrode(s) on the basket catheter exhibiting earliest activation on raw data. The electrode identified as a leader on the STAR maps was also the closest basket electrode to the pacing site when reviewing the CARTO3 maps.

### 4.3 AT validation

STAR mapping was able to identify both macro‐reentrant and focal/micro‐reentry mechanisms. Macro‐reentrant mechanisms are depicted by arrows showing a continuous direction of activation, with no electrodes leading all the time, but differences in the proportion of the time an electrode lead depending on which electrodes it is paired to locally. Focal/micro‐reentry are depicted by the nearest electrode consistently leading all others, with surrounding electrodes leading less and less often compared to neighboring electrodes with greater distance from the source. Arrows also show the direction of the wavefront away from this leading electrode. The purpose of mapping AT was partly to assess this novel‐mapping method in terms of its ability to correctly discern wavefront propagation before applying the method to AF. However, the STAR mapping method could have a role in mapping AT. In contrast to conventional LAT mapping, the STAR mapping method is not dependent on a fixed reference. Thereby with an AT that demonstrates a changing CL, as is common for focal AT or localized reentry in scarred atria, the STAR mapping method could have advantages over conventional activation mapping since activation times are instead compared in a dynamic fashion across electrode pairs.

An important consideration in STAR mapping is the limitations on GEPD which is perhaps analogous to interpolation on conventional activation mapping. The ability to alter the GEPD allows the STAR mapping method to be adaptable. For regular macro‐reentrant circuits, this distance can be increased to allow pairing of electrodes over larger distances. Pairing electrodes over larger distances will pair electrodes across unmapped areas, for example in roof dependent tachycardia electrodes might be paired across gaps commonly left by the basket catheter at the roof or septum. However, the greater this distance is increased, the greater the potential for error, particularly with focal activation. Conversely, very short GEPD will result in several small islands on the LA surface with a lead edge to each which then has to be considered as a whole and analyzed with care.

Using a GEPD greater than 6 cm results in a large proportion of electrode pairs that map different anatomical surfaces, for example, posterior and anterior LA wall, to be paired with each other. Although this might be acceptable for a macro‐reentrant AT with a slow CL, this could create misleading STAR maps in other situations, and therefore GEPD this long should be avoided. Using GEPD of 3 cm resulted in the consistent mapping of larger anatomical surfaces without breaking them into small islands, but also did not join anatomical surfaces separated by large gaps.

Although these settings worked well for mapping of ATs, it is conceivable that the GEPD may have to be reduced for disorganized rhythms. STAR mapping was conceived with the aim that it might be used to map AF. Studies have shown that localized drivers of AF show a degree of spatial stability and temporal periodicity.^3^, ^12^, ^13^ Determining the propensity of an electrode to lead relative to its neighbors a high proportion of the time (even if not consistently) may allow identification of such spatially conserved but intermittent drivers. Localized drivers of AF are also thought to occupy a small area of the LA.^1^, ^2^ The flexibility of the GEPD may facilitate identification of drivers where only a relatively small area of the LA responds to the driver in a 1:1 activation pattern.

There are several mapping systems/methods currently being explored for their role in mapping drivers in AF. The STAR mapping method identified leading sites of activation which were localized using a replica of the three‐dimensional geometry created using CARTO3. This thereby aids the operator when they navigate to ablation target sites. This is in contrast to the Topera mapping system (Abbott, Menlo Park, CA)^15^ that relies on a 2D animation of the geometry, or the ECGi mapping system (CardioInsight, NonInvasive 3D mapping system, Medtronic, Minneapolis, MN) 13 that uses a geometry obtained from CT imaging data.

CARTO, precision, and rhythmia are all capable of conventional activation mapping, although this is of limited usefulness in AF. Data suggests that electrogram characteristics, fractionation, and frequency analysis all correlate poorly with drivers in AF. CARTO has recently produced CARTOFINDER (Biosense Webster) which times electrograms relative to each other in a 250 ms window which then moves through a continuous recording to show wavefront movement over time. This has enjoyed some success demonstrating focal and rotational activations in AF and an automated approach to their detection has been developed.^3^, ^12^, ^16^ The Topera and ECGi mapping systems rely on phase mapping to identify drivers. These technologies have also enjoyed some success; although there has also been concern that phase mapping could demonstrate rotors incorrectly at times.^17^ In contrast, the STAR mapping method utilizes minimal computation or data manipulation and simply compares activation times across electrode pairs to identify sites that are most often leading compared to neighboring sites. Furthermore, all currently available mapping systems require a degree of interpretation by the operator, such as for analysis of phase maps with the ECGI, or analysis of dynamic wavefront activation maps with CARTOFINDER. In contrast, the STAR mapping method is not dependent on the analysis of dynamic wavefront maps but works through highlighting the ablation target sites by a colored circle (circle color is representative of the time an electrode is leading relative to its pairs). The STAR mapping methodology, therefore, has some potential advantages for mapping in AF and clinical studies are underway investigating this (NCT029508844).

### 4.4 Limitations

This was a single center study in which experienced operators that developed the method and know it well used the mapping method. Nevertheless, the analysis of whether STAR maps correlated with the sites of atrial paced beats or AT was repeated offline by observers not involved with the procedure who achieved an identical result.

The aim of this study was to validate the STAR mapping method by correlating its outputs with known arrhythmia mechanisms, i.e. pacing, AT and calcium transit on a HL1 cell array. Further clinical studies are therefore necessary to establish whether STAR mapping provides clinically useful information that facilitates the mapping of other arrhythmias. Further validation of the method in the setting of AF is also required.

## 5 CONCLUSIONS

The STAR mapping method is a novel‐mapping method that has been effectively validated using an in vitro model, in addition to mapping during LA pacing and AT in vivo. The mapping method effectively demonstrated focal/micro‐reentrant and macro‐reentrant ATs in the RA and LA. Furthermore, the offline analysis showed that AT mechanisms could be determined using STAR maps alone, suggesting that it might be useful as an adjunctive method for mapping AT. This mapping method may have the potential for mapping disorganized rhythms such as AF.

## Supporting information

Supporting information

Supporting information

Supporting information
