# Supplementary material for: Development, in vitro validation and human application of a novel method to identify arrhythmia mechanisms: The stochastic trajectory analysis of ranked signals mapping method
Source: J Cardiovasc Electrophysiol. 2019 Mar 5;30(5):691–701. doi: 10.1111/jce.13882 (PMC8609431; doi:10.1111/jce.13882)
Supplement: Supplementary file 1 — Supporting information [file JCE-30-691-s003.docx]

SUPPLEMENTAL METHOD

*A) In vitro study*

1. *Pre-coating flasks*

Prior to culturing, the HL1 cells onto flasks these were pre-coated with a gelatin/fibronectin coat (Sigma-Aldrich, Inc, US). The gelatin/fibronectin coating was made through initially weighing out 0.1g of gelatin. This was placed into a 500ml glass bottle and distilled water was added to the 500ml mark. This was then autoclaved until the gelatin dissolved, making a gelatin concentration of 0.02%. The fibronectin was then added to the gelatin suspension whereby 1ml of fibronectin was diluted in 199ml of 0.02% gelatin. Immediately following this, aliquoted 6ml aliquots were placed into 15ml centrifuge tubes. The aliquots were then frozen at -20 degrees. The flasks (T75) were then coated with 3ml of gelatin/fibronectin coating prior to culturing. The flasks were then capped and incubated at 37°C for one hour.

1. *Culturing cells*

The coated T75 flasks were then used to culture the cells by adding 14ml of supplemented Claycomb medium^^[[1]](#footnote-1)^^ (growth medium) followed by 1ml of HL1 cells making up a total solution of 15ml.

The cells were assessed daily to ensure continued growth with the supplemented Claycomb medium changed daily. Once the cells reached full confluence, passaging was performed with a 1:3 split.

1. *Passaging with 1:3 split*

Firstly, the flask was rinsed with 3ml of warmed phosphate buffered saline by pipetting the solution to the base of the flask then rinsing gently and then removing the solution by aspiration. To separate the cells from the flask, 3ml of trypsin/EDTA (0.05% trypsin in 0.02% EDTA-Na) was added to the flask and intubated for 1min at 37°C. This was then repeated by removing and adding 3ml of fresh trypsin/EDTA solution and incubating for an additional 2 minutes at 37°C. The flask was then examined microscopically to ensure the cells were no longer adherent to the flask. If there were cells thought to be adherent the flask was gently tapped to dislodge the remaining cells. To inactivate the enzyme, 3ml of supplemented Claycomb medium was added to the cells. The cells were then transferred from the flask onto a 15ml centrifuged tube. To ensure all cells were removed, the flask was washed with 5ml of the supplemented Claycomb medium. This was then aspirated and added to the cells in the 15ml centrifuged tube. This was then centrifuged at 500xg for 5 minutes (Eppendorff Hamburg, Germany). The supernatant was then removed by aspiration and the pellet re-suspended in 3ml of supplemented Claycomb Medium, out of which 1ml was added to each of the three gelatin/fibronectin coated flasks together with an additional 14ml of supplemented Claycomb Medium.

1. *MEA (multi-electrode arrays) plates and HL1 experiments*

During splitting, a proportion of the cells were placed onto MEA plates (300 microliters cell suspension and 700 microliters supplemented Claycomb Medium). The MEA plates used were standard plates consisting of 60 flat round titanium nitride electrodes in an 8x8 layout grid. The interelectrode spacing distance was 100 μm with electrode diameters of 10 μm.

These were used to map spontaneous electrical activity and calcium transit through optical mapping. This was performed at 48h, because in our experience use of the cells prior to this meant that the cells on the plates were not confluent and later than 48h resulted in cell death and differentiation into fibroblasts limiting the mapping of electrical activity.

Prior to performing the experiments, a buffer was created using 90ml of distilled water, 10ml of Kreb-Henseleit solution and 140micrograms of Calcium chloride. This was then oxygenated. The Claycomb medium was then removed from the MEA plates and replaced with the oxygenated buffer.

1. Supplemented Claycomb Medium- Claycomb Medium 87ml, Fetal Bovine Serum 10ml, Pencillin/Streptomycin 1ml, Norepinephrine (10mM stock) and L-Glutamine (200mM stock) 1ml [↑](#footnote-ref-1)
