# Supplementary material for: Development, in vitro validation and human application of a novel method to identify arrhythmia mechanisms: The stochastic trajectory analysis of ranked signals mapping method
Source: J Cardiovasc Electrophysiol. 2019 Mar 5;30(5):691–701. doi: 10.1111/jce.13882 (PMC8609431; doi:10.1111/jce.13882)
Supplement: Supplementary file 2 — Supporting information [file JCE-30-691-s001.docx]

***Supplemental Table 1-*** *Baseline characteristics*

| **Baseline characteristics** | | **Cohort n=25** |
| --- | --- | --- |
| Age years. mean ± SD | 62.2±10.7 | |
| Male n (%) | 18 (72.0) | |
| Diabetes mellitus n (%) | 1 (4.0) | |
| Hypertension n (%) | 8 (32.0) | |
| TIA/CVA^*^ n (%) | 1 (4.0) | |
| Ischemic heart disease n (%) | 2 (8.0) | |
| Cardiac surgery n (%) | 1 (4.0) | |
| Left ventricular EF^+^ ≥ 55% n (%) | 21 (84.0) | |
| LA size cm^2^ n (%)  20-30  30-40  >40 | 18 (72.0)  6 (24.0)  1 (4.0) | |
| Persistent AF n (%) | 10 (40.0) | |
| Previous AF ablation (out of the 15 AT patients) n (%)  WACAs^§^  Complex fractionated atrial electrograms  Roof line  Mitral isthmus line | 13 (86.7)  13 (86.7)  3 (23.1)  5 (38.5)  6 (46.1) | |
| Previous AT ablation (including AT and AF patients) n (%)  Cavo-tricuspid isthmus-dependent flutter  Focal/micro-reentrant | 9 (36.0)  2 (8.0) | |
| Current medical strategy n (%)  Beta-blockers including Sotalol  Amiodarone  Flecainide | 17 (68.0)  7 (28.0)  4 (16.0) | |

^*^TIA/CVA- Transient ischemic event/Cerebrovascular accident

^+^EF- Ejection fraction

^§^WACAs- Wide area circumferential ablation
