# Supplementary material for: Development, in vitro validation and human application of a novel method to identify arrhythmia mechanisms: The stochastic trajectory analysis of ranked signals mapping method
Source: J Cardiovasc Electrophysiol. 2019 Mar 5;30(5):691–701. doi: 10.1111/jce.13882 (PMC8609431; doi:10.1111/jce.13882)
Supplement: Supplementary file 3 — Supporting information [file JCE-30-691-s002.docx]

***Supplemental Table 2-*** *Demonstrates the proportion of the surface area of the anatomical geometry over which pole pairing was performed with a range of geodesic distances.*

| **Geodesic distance, cm** | **Area covered, cm^2^** | **Number of paring segments**  **Anterior Septal Roof Posterior Lateral** | **Proportion of pole pairs that only include poles that see the same wavefront i.e. map the same anatomical surface %** |
| --- | --- | --- | --- |
| 1 | 0.7 ± 0.5 | 4.6 ± 1.1 2.4 ± 1.1 3.0 ± 1.6 3.7 ± 1.0 2.8 ± 1.5 | 100 |
| 2 | 1.2 ± 0.3 | 3.3 ± 0.9 1.9 ± 0.7 2.4 ± 0.5 3.0 ± 0.6 2.3 ± 1.0 | 100 |
| 3 | 1.8 ± 0.3 | 2.5 ± 0.5 1.5 ± 0.5 2.1 ± 0.4 2.7 ± 0.5 1.8 ± 0.8 | 100 |
| 4 | 3.3 ± 0.5 | 2.2 ± 1.2 1.4 ± 0.5 1.3 ± 0.5 2.2 ± 0.8 1.2 ± 0.4 | 100 |
| 5 | 5.2 ± 1.1 | 1.5 ± 1.1 1.1 ± 0.4 1.0 ± 0 1.3 ± 0.5 1.1 ± 0.4 | 100 |
| 6 | 6.8 ± 1.5 | 1.5 ± 1.1 1.0 ± 0 0.9 ± 0.4 1.0 ± 0.5 0.9 ± 0.4 | 82.5 |
| 7 | 7.2 ± 1.7 | 1.5 ± 1.2 1.0 ± 0 1.1 ± 0.7 1.1 ± 0.4 0.7 ±0.5 | 37.5 |
| 8 | 8.4 ± 2.1 | 1.1 ± 1.2 0.6 ± 0.5 0.9 ±0.9 1.0 ± 1.0 0.6 ± 0.5 | 0 |
| 9 | 8.8 ± 2.4 | 1.0 ± 1.3 0.5 ± 0.5 0.4 ± 0.5 0.6 ± 0.8 0.4 ± 0.5 | 0 |
